# Clinical Examination Findings Can Accurately Diagnose Developmental Dysplasia of The Hip—A Large, Single-Center Cohort

**DOI:** 10.3390/children10020304

**Published:** 2023-02-04

**Authors:** İzzet Özay Subaşı, Enejd Veizi, Şahin Çepni, Hilmi Alkan, Temel Oğuz, Ahmet Fırat

**Affiliations:** 1 Department of Orthopedics and Traumatology, Faculty of Medicine, Erzincan University, Erzincan 24100, Turkey; 2 Department of Orthopedics and Traumatology, Ankara City Hospital, Ankara 06000, Turkey

**Keywords:** developmental dysplasia of the hip, skin creases, limited hip abduction, ultrasound, infant

## Abstract

Background: Physical examination findings such as limited hip abduction (LHA), asymmetric skin creases (ASC), and a popping sensation in the hip facilitate the diagnosis of developmental dysplasia of the hip (DDH). Screening with a simple physical examination during the first weeks of infancy is important for early detection of the condition, and a wide range of medical professionals, including general practitioners, obstetricians, pediatricians, and orthopedic surgeons etc. are involved in this process. The aim of this study was to determine the correlation between easily recognizable physical examination findings such as LHA, thigh/groin ACSs, and Ortolani and Barlow tests with ultrasound findings for the diagnosis of DDH. Methods: This study included 968 patients undergoing routine hip ultrasonography between December 2012 and January 2015. All patients were examined by an experienced orthopedic surgeon who was not the physician who performed the ultrasound examination to exclude bias between physical examination findings and ultrasound findings. Asymmetric skin folds (thigh and groin), limited abduction, Barlow and Ortolani tests were recorded. The relationship between the physical examination findings, ultrasound findings, and developmental dysplasia was investigated. Results: Of the 968 patients, 523 were female (54%) and 445 were male. On ultrasonography examination, 117 patients were found to have DDH. The sensitivity, specificity and negative predictive values of patients who were found to have both LHA and thigh/groin ASCs in all three physical examinations were high (83.8%, 70.2%, and 96.9%, respectively) while positive predictive values were found to be low (27.8%). Conclusion: Asymmetric skin creases on the thigh and groin and limited hip abduction, when evaluated together, have high sensitivity and specificity with additional high negative predictive values and could help during the initial screening process of DDH.

## 1. Introduction

Developmental dysplasia of the hip (DDH) is a common musculoskeletal disorder affecting children. This condition refers to a series of abnormalities of the hip joint, including neonatal instability, acetabular dysplasia, hip subluxation, and true dislocation of the hip [1,2,3]. The risk factors include a positive family history, breech presentation, swaddling, first-born infant, oligohydramnios, multiple pregnancies, and female gender [3,4,5,6,7], while genetic factors have only recently been described [8]. Two main theories have been developed regarding the etiology of DDH [2]. Hormonal theory emphasizes the imbalance between estrogens and progesterone but although this theory has some support, no relationship between DDH and serum concentration of beta-estradiol and relaxine has yet been demonstrated [9]. The anatomical development of the hip joint requires concentric positioning of the femoral head into the acetabular cavity and adequately balanced growth between the triradiate and acetabular cartilage, and this has led to the proposition of a mechanical theory that persistent mechanical stimulation provokes deformity. The maintained intrauterine posture of forced hyperflexion of the hip and extension of the knee associated with breech presentation is suspected to lead to hip dysplasia and subsequent dislocation [10].

It is challenging to determine the exact incidence of DDH, with the condition probably being underreported. The literature offers a range between 0.1% and 10% of live births, and female sex predominates [1,11]. Treatment depends on several factors such as time of detection, additional neurological or developmental conditions and access to medical care [3]. The majority of cases are treated conservatively with a Pavlic harness. Closed and open surgical reduction is generally reserved for cases not responding to conservative treatment or late presentation. Osteotomies are generally performed after the 18th month of life [12]. If the diagnosis of DDH is overlooked or missed, it leads to increased morbidity and functional limitations such as leg length discrepancies, gait abnormalities, limited range of motion, and pain [4,7,13,14]. Early detection, however, allows the opportunity for complete healing with no to minimal adverse effects [15].

Screening for DDH with physical examination is the first step towards recognizing the condition. Limited hip abduction (LHA), asymmetric skin creases (ASCs), and a popping sensation in the hip (Barlow and Ortolani tests) can help during diagnosis [12]. These findings may prompt the inexperienced attending physician to refer the patient to an orthopedic surgeon [13,16,17,18]. The Barlow and Ortolani tests are important findings usually present during physical examination, but they can sometimes be challenging to perform [19]. Although the findings of this physical examination are helpful, they may not be sufficient alone to diagnose DDH in an infant within the first six months of life. [18,20]. The current gold standard for a diagnosis of DDH is a hip ultrasound performed according to the method described by Graf [21,22,23,24,25].

There is still a lack of agreement regarding whether there is a correlation between the findings of a physical examination and those of an ultrasound when diagnosing DDH. Although ASCs in the groin and thigh regions are generally accepted as an early clinical sign of DDH, there are publications in the literature that state the opposite and discourage the use of ASCs for screening purposes [16,26]. The definition of asymmetry and the lack of consensus regarding which ASCs should be considered abnormal are two of the primary factors that have contributed to this debate [17,27,28]. Since screening by physical examination is often performed by physicians other than orthopedic surgeons, a correlation between these findings and DDH diagnosis becomes important. Therefore, the aim of this study was to determine the correlation of easily recognizable physical examination findings such as LHA, thigh/groin ACSs and Ortolani and Barlow tests with ultrasound findings for the diagnosis of DDH in a large single-center cohort.

## 2. Materials and Methods

All study procedures were performed in accordance with the 1964 Declaration of Helsinki and all its subsequent amendments. The study design was approved by our institutional review board.

### 2.1. Patient Selection

This retrospective cohort study included patients who presented at our medical facility for a physical and ultrasonographic examination of DDH between December 2012 and January 2015. Since 2010, a nationwide active screening program has been implemented in Turkey with a hip ultrasound for all infants between 1.5 and 6 months [4,23]. All mothers are advised and referred by their respective family doctor to an orthopedic surgeon for a routine screening between 1.5 and 2 months after birth. Inclusion criteria for this study were detailed notes regarding physical examination with an emphasis on the presence of ASCs, LHA and results of Barlow and Ortolani tests, parental approval and ultrasound results obtained on the same day as the physical examination. Patients were excluded from the study if hips were diagnosed as neuromuscular or teratological, if the hips had been initially treated at another center, or if parental consent was not provided.

A total of 968 patients were included in the final analysis. The mean age of the infants at the time of presentation was 10.4 ± 4.64 weeks with 95.5% of patients being within the range advised by the screening program (1.5–2-months-old). The study population consisted of 523 females (54%) and 445 male (46%) infants.

### 2.2. Data Gathering

A detailed medical history was obtained from the parents. All infants were examined by one of three experienced pediatric orthopedic surgeons with gentle manipulations at an appropriate room temperature within 30 min of breastfeeding. Following the physical examinations, ultrasonography was performed by another pediatric orthopedic surgeon experienced in ultrasonography of the pediatric hip and who was blinded to the findings of the physical examination of all infants to avoid bias between the results of the physical examination and the ultrasound outcomes. All data were collected prospectively.

**Figure 1:**
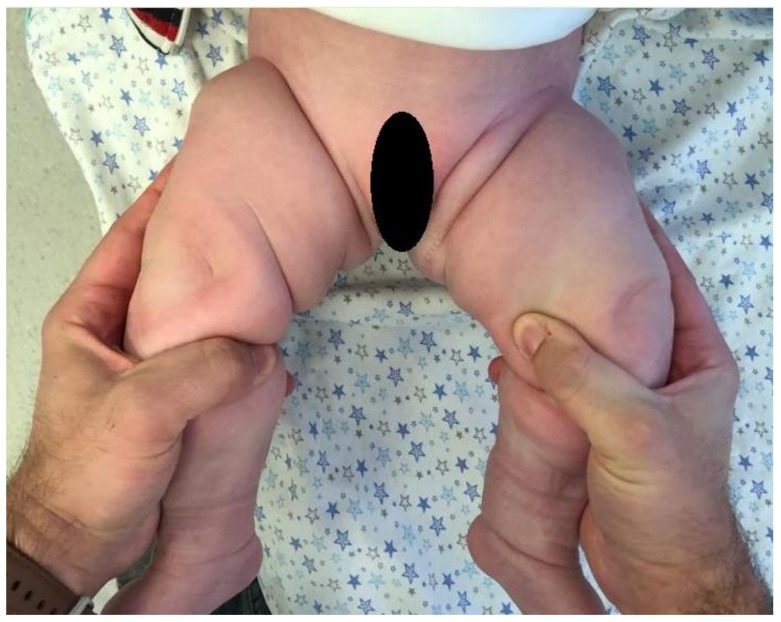
Anterior asymmetric skin creases during a Barlow and Ortolani test.

**Figure 2:**
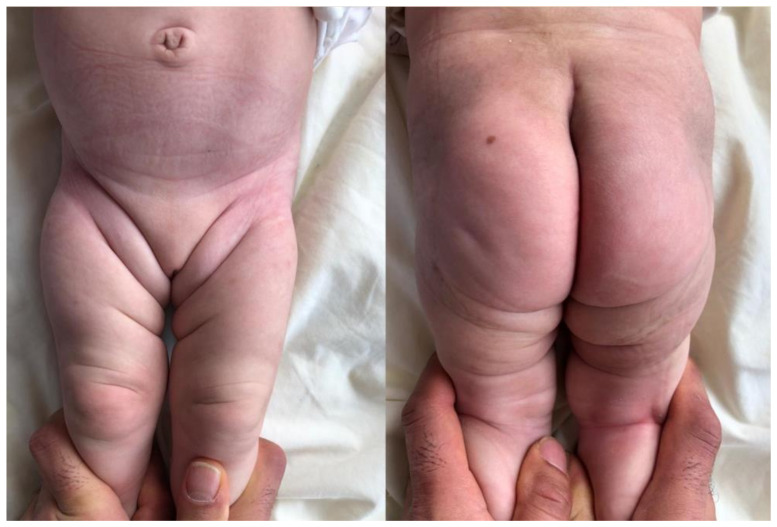
Symmetric skin creases of a patient with bilateral hip dysplasia.

The results of the ASCs (both in the groin and the thigh), LHA, Barlow, and Ortolani tests were recorded. (Figure 1). When assessing ASCs, it was ensured that the hips were in an adducted position and that the knee and hip joints were in a fully extended position. During the examination, separate assessments of ASCs in the groin and thighs were conducted. (Figure 2). When unilaterally LHA was evaluated, limitations greater than 20 degrees compared with the other hip were considered significant [29,30]. In order to conduct a clinical evaluation of LHA, both hips were flexed to an angle of ninety degrees and an effort was made to achieve full abduction in both joints. A goniometer was used to assess bilateral LHA. The Barlow and Ortolani tests were performed only once in the infants. The hips that were positive on one of the Barlow and Ortolani tests were evaluated as clicky hips [19].

**Figure 3:**
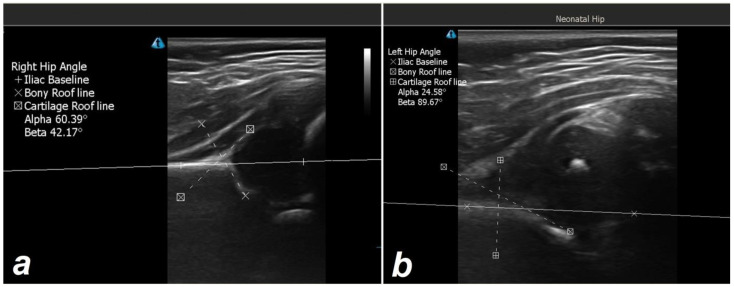
Ultrasonic examination of a patient without (**a**) and with developmental hip dysplasia (**b**).

All ultrasound examinations were performed on a special table and in the lateral decubitus position by an experienced pediatric orthopedic surgeon (Figure 3). It was routine to make use of a linear probe with a frequency response of 7.5 mm/Hz (Fukuda FFsonic UF-4100, Fukuda Denshi Co., Ltd., Tokyo, Japan). On the images printed from the hip ultrasound, a goniometer was used to perform angle measurements. A morphological measurement of the hip known as the Graf alpha angle was utilized. The alpha angle of a normal hip is greater than 60 degrees, while the alpha angle of a Graf type-II dysplastic hip ranges from 43 degrees to 60 degrees, and the alpha angle of a Graf type-III dysplastic hip is less than 43 degrees. Every child’s hip was scanned while flexed and adducted. This reduced the likelihood of errors brought on by pelvic obliquity, which could have resulted in an incorrect interpretation of the scan. Hips that were either dislocated or capable of becoming dislocated were classified as type IV hips [25,29]. Infants determined with DDH (Graf types IIa (-), IIb, IIc, D, III, IV) were designated as Group 1 and infants with normal ultrasonographic examinations as Group 2 according to the ultrasonographic evaluation based on the Graf method [22,25].

### 2.3. Statistical Analysis

The data obtained in the study were analyzed statistically using SPSS v. 22.0 software (IBM Corpn., Armonk, NY, USA). Pearson Chi-square and Fisher’s Exact tests were used to compare categorical data to independently assess relationships between sex, age, physical examination findings, and DDH. The Mann-Whitney U-test was used to compare mean values between groups. Sensitivity, which quantifies a test’s ability to detect true positives, and specificity, which quantifies a test’s ability to detect true negatives, were calculated. The positive and negative predictive values of the study variables (ASCs, LHA and Ortolani/Barlow) to diagnose DDH were also calculated. The sensitivity, specificity, positive predictive, and negative predictive values of the physical examination results were compared with those of the ultrasonography results. The level of statistical significance for each test was set as *p* ≤ 0.05. Post-hoc power analysis using the G*Power 3.1 program (Düsseldorf University in Düsseldorf, Germany) determined that the study had power of 99%.

## 3. Results

Of the total study cohort, 117 (12.1%) infants could be categorized as Graf type IIa (-), IIb, IIc, D, III, or IV. The results of the ultrasound examinations showed that 58 infants (49.6%) had DDH in the left hip, 43 infants (36.8%) had DDH in the right hip, and 16 infants (13.6%) had DDH in both hips. Of the infants diagnosed with DDH, 98 (83.8%) were female, and 19 (16.2%) were male. A statistically significant difference was found between patient gender and DDH (*p* ˂ 0.001).

A statistically significant difference between Groups 1 and 2 was also obtained for all of the physical examination elements. Positive Barlow and Ortolani findings were noted only in Group 1, diagnosed with DDH. As stated, due to neuromuscular disease or teratological hips, all patients whose dislocated hips were irreducible (Ortolani negative) were initially excluded from the study. Patients with ASCs of the thigh and the groin and LHA were present in both study groups. The associations between physical examination findings and DDH are shown in Table 1.

The sensitivity, specificity, and negative and positive predictive values of physical examination findings when evaluated alone are listed in Table 2. While having high specificity for DDH, the physical examination findings alone showed low sensitivity and positive predictive values.

The sensitivity, specificity, negative predictive, and positive predictive values of infants with at least two or three positive physical examination findings for LHA, thigh and groin ASCs are presented in Table 3. When used in combination with each other, all three physical examination findings were found to have high sensitivity, specificity, and negative predictive values.

## 4. Discussion

The most important finding of this study was that groin and thigh ASCs together with a LHA have high sensitivity, specificity, and negative predictive value, while the positive predictive value is low. Another critical finding of the study was the low sensitivity of the Barlow and Ortolani tests, despite high specificity, positive and negative predictive values.

In the literature, DDH incidence has been reported at rates up to 10% [1,16,31,32]. In the current study, the ultrasonography results determined DDH in 117 patients (12.1%), which was a slightly higher rate than reported in previous studies. This can be attributed to the fact that our hospital is a referral hospital for DDH. It has been previously shown that the risk of developing DDH is 9-fold greater in females than in males [5]. Similarly, the current research revealed that the incidence of DDH in females was noticeably higher. DDH affects the left hip more frequently than it does the right hip, and 20% of cases involve both hips [5]. The findings of the current study showed bilateral DDH in 16 infants (13.6%), 58 infants (49.6%) had DDH in the left hip, and 43 infants (36.8%) had DDH in the right hip. The reason for this is thought to be due to the intrauterine position of the fetus.

DDH is a common musculoskeletal disorder in childhood [1,16]. Determining risk factors, history, findings from a physical examination, and imaging techniques all play an essential part in diagnosing DDH. It is not possible to perform an ultrasound examination on every infant due to the high cost of medical care, the limited availability of trained personnel, and the organizational challenges involved [29,30]. Therefore, the findings of a physical examination are important in determining the presence of DDH. Even though an accurate diagnosis and timely treatment of DDH can result in favorable clinical outcomes, DDH that is either not treated or is treated incorrectly is a significant contributor to morbidity [4,17,33]. Experience is required for the evaluation of the Barlow and Ortolani tests. In contrast, the findings of a physical examination, such as ASCs and LHA, can be recognized by less experienced physicians with greater ease [19,33]. These easily recognized physical examination findings are important in referring suspicious infants to pediatric orthopedists for definitive diagnosis and treatment of DDH.

The hips with positivity determined in one of the Barlow and/or Ortolani tests were called clicky hips. Many factors, such as soft tissue ligaments, can contribute to the development of a clicky hip by disrupting the typical relationship that exists between the acetabulum and the femoral head. In a large retrospective cohort study that examined 7864 newborns, clicky hip was determined in 622 (8%) newborns by inexperienced physicians. Follow-ups of these newborns by pediatric orthopedic surgeons revealed hip pathology requiring treatment in only 34 (0.5%) newborns [19]. This demonstrates that the clicky hips detected by physicians with a lack of experience can be deceiving [19,34]. In the current study, only 9 (0.9%) of 968 infants were positive for Barlow and Ortolani tests. There were no Ortoloni-negative hips in this study, as all the patients with irreducible dislocated hips (Ortolani-negative) were initially excluded from the study due to neuromuscular disease or teratological hips. Although all the infants with clicky hips were found to have DDH (specificity 100%), the sensitivity of Barlow and Ortolani tests was 2.6% and 5.1%, respectively. This indicates that the detection of a clicky hip in order to diagnose DDH is an extremely uncommon occurrence. Clicky hips detected by physicians with limited experience could increase healthcare costs with unnecessary referrals of healthy infants to pediatric orthopedists.

In infants suspected of having DDH, an ASC may be one of the first findings discovered during a physical examination [4,33]. The proximal displacement of the femoral head and the compression of the skin in a hip that is subluxated or dislocated are the two factors responsible for forming ASCs [17]. ASCs are easily observed findings that are important in evaluating DDH. Groarke et al. [16] assessed the thigh and groin ASCs together in their study and found the sensitivity, specificity, and positive predictive values to be low (46.2%, 42.6%, and 12.4%, respectively) and the negative predictive value to be high (81.8%). The aforementioned related values were higher in the current study (sensitivity 63.3%, specificity 77.3%, positive predictive value 27.7%, and negative predictive value 93.9%). As our hospital is a tertiary hospital for the pediatric population, the doctors who perform the physical examination are more experienced in DDH than the practitioners who refer patients to us.

In a study by Sevencan et al. [15], the asymmetrical distribution of skin creases was investigated separately in each of the three regions (inguinal, gluteal, and thigh). Hip ultrasonography was then performed, and the Graf method was applied to categorize the cases as either “centralized” (Graf types I, IIa (-), IIb, and IIc) or “decentralized” (Graf types IId, III, and IV). Both univariate and multivariate analyses were used to investigate and evaluate the nature of the relationship between the groups. The presence of ASC in the inguinal and gluteal regions was reported to increase the risk of the child developing a decentralized hip, but the presence of ASC in the isolated thigh region did not increase the risk of developing a dislocated hip. It was also speculated that the presence of ASC in the inguinal and gluteal regions are reliable findings in DDH screening, although a statistical correlation between isolated thigh ASC and DDH could not be established in that study. In parallel with that study, the sensitivity of the ASCs located in the groin and thighs was determined to be low in the current research. Nevertheless, the combination of these findings brought the level of sensitivity up to a point that was satisfactory.

In addition to being a significant finding for DDH in a physical examination, LHA is a finding that is frequently seen in hips that are decentralized [30,35]. In a study by Jari et al. all (100%), decentralized hips were found to be positive for LHA [35]. Similarly, LHA was observed in 20.6% of infants (n = 24) diagnosed with DDH in the current study. Of these 24 infants diagnosed with DDH, 9 had a decentralized hip on ultrasonography, and all 9 patients (100%) had LHA. A statistically significant association was determined between the DDH and LHA (*p* ˂ 0.001).

Comparing the findings of ultrasonography with those of physical examination, which is the most valuable diagnostic method in DDH, has been the focus of a number of studies published with the intention of contributing to clinical practice [4,16,24,36,37]. Omeroğlu et al. [17] reported that ASCs alone increase the risk of DDH by 3.5-fold, but an additional positive physical examination finding such as Ortolani/Barlow test or LHA in addition to ASCs may increase the risk by 7.5-fold. In the current study, it was concluded that although the sensitivity and specificity of these physical examination findings alone were weak, the co-occurrence of thigh and groin ASCs and LHA findings increased the sensitivity and specificity. When these three physical examination findings were evaluated together, the sensitivity increased to 83.8%, specificity to 70.2%, and negative predictive value to 96.9%. In contrast, no significant increase in positive predictive value was detected in this study. According to the results obtained in this study, physical examination findings that can be easily recognized by physicians with limited experience are quite successful in diagnosing DDH when they are evaluated together. In addition, the specificity, and positive and negative predictive values of Ortolani and Barlow tests, which are difficult for practitioners or family doctors to perform, were high in experienced hands.

There were some limitations to this study. Although three different orthopedic surgeons performed the physical examinations of the infants, they all had at least five years of experience in pediatric orthopedics. The sensitivity, specificity, and positive/negative predictive values may have been higher than those of other studies because our hospital is a tertiary referral hospital, the study population was large, and the physicians who performed the physical examination were experienced pediatric orthopedic surgeons.

## 5. Conclusions

Physical examination findings such as ASCs and LHA may result in low sensitivity, specificity, positive and negative predictive values when assessed alone. By combining these easily recognizable findings from an infant’s physical examination, the likelihood of diagnosing DDH increases significantly. Asymmetric skin creases on the thigh and groins and limited hip abduction, when evaluated together, have high sensitivity, specificity and negative predictive values with additional low positive predictive value and could help during the initial screening process of DDH.

## Figures and Tables

**Table 1:** Relationships between examination findings and developmental dysplasia of the hip.

	Group 1 (n = 117)	Group 2 (n = 851)	*p* *
Barlow	3 (2.5%)	-	<0.05
Ortolani	6 (5.1%)	-	<0.001
Thigh ASCs	43 (37.3%)	129 (15.2%)	<0.001
Groin ASCs	31 (26.5%)	64(7.6%)	<0.001
LHA	24 (20.6%)	61 (7.2%)	<0.001

ASCs: asymmetrical skin creases, LHA: limited hip abduction. * Mann-Whitney U test.

**Table 2:** Sensitivity, specificity, positive predictive, and negative predictive values of physical examination findings of the patients.

	Sensitivity	Specificity	PPV	NPV
Barlow	2.6%	100%	100%	88.2%
Ortolani	5.1%	100%	100%	88.5%
Thigh ASC	36.8%	84.8%	25%	90.7%
Groin ASC	26.5%	92.5%	32.6%	90.2%
LHA	20.5%	92.8%	28.2%	89.5%

ASCs: asymmetrical skin creases, LHA: limited hip abduction, PPV: positive predictive value, NPV: negative predictive value.

**Table 3:** Combination of the sensitivity, specificity, and negative and positive predictive values of patients with at least two or three positive physical examination findings of LHA, thigh ASCs, and groin ASCs.

	Sensitivity	Specificity	PPV	NPV
Thigh ASCs + Groin ASCs	63.3%	77.3%	27.7%	93.9%
Thigh ASCs + LHA	57.3%	77.7%	26.1%	93%
Groin ASCs + LHA	47%	85.3%	30.6%	92.1%
Thigh ASCs + Groin ASCs + LHA	83.8%	70.2%	27.8%	96.9%

ASCs: asymmetrical skin creases, LHA: limited hip abduction, PPV: positive predictive value, NPV: negative predictive value.

## References

[1] Young J.R., Anderson M.J., O’Connor C.M., Kazley J.M., Mantica A.L., Dutt V. (2020). Team Approach: Developmental Dysplasia of the Hip. JBJS Rev..

[2] Vaquero-Picado A., González-Morán G., Garay E.G., Moraleda L. (2019). Developmental dysplasia of the hip: Update of management. EFORT Open Rev..

[3] Canavese F., Castañeda P., Hui J., Li L., Li Y., Roposch A. (2020). Developmental dysplasia of the hip: Promoting global exchanges to enable understanding the disease and improve patient care. Orthop. Traumatol. Surg. Res..

[4] Ömeroğlu H., Akceylan A., Köse N. (2019). Associations between risk factors and developmental dysplasia of the hip and ultrasonographic hip type: A retrospective case control study. J. Child. Orthop..

[5] Agarwal A., Gupta N. (2012). Risk factors and diagnosis of developmental dysplasia of hip in children. J. Clin. Orthop. Trauma.

[6] Terjesen T., Horn J. (2020). Prognostic value of severity of dislocation in late-detected developmental dysplasia of the hip. J. Child. Orthop..

[7] Merchant R.M., Tolk J.J., Ayub A.A., Eastwood D.M., Hashemi-Nejad A. (2022). The Importance of Monitoring and Factors That May Influence Leg Length Difference in Developmental Dysplasia of the Hip. Children.

[8] Harsanyi S., Zamborsky R., Kokavec M., Danisovic L. (2020). Genetics of developmental dysplasia of the hip. Eur. J. Med. Genet..

[9] Andersson J.E., Vogel I., Uldbjerg N. (2002). Serum 17 beta-estradiol in newborn and neonatal hip instability. J. Pediatr. Orthop..

[10] Vaquero-Picado A., Moraleda L., Forriol Campos F. Validation of an experimental model of developmental dysplasia of the hip. Proceedings of the 35th EPOS Annual Meeting.

[11] Vasilcova V., AlHarthi M., AlAmri N., Sagat P., Bartik P., Jawadi A.H., Zvonar M. (2022). Developmental Dysplasia of the Hip: Prevalence and Correlation with Other Diagnoses in Physiotherapy Practice-A 5-Year Retrospective Review. Children.

[12] Pavone V., de Cristo C., Vescio A., Lucenti L., Sapienza M., Sessa G., Pavone P., Testa G. (2021). Dynamic and Static Splinting for Treatment of Developmental Dysplasia of the Hip: A Systematic Review. Children.

[13] Stein-Zamir C., Volovik I., Rishpon S., Sabi R. (2008). Developmental dysplasia of the hip: Risk markers, clinical screening and outcome. Pediatr. Int..

[14] Lim C., Cho T.-J., Shin C.H., Choi I.H., Yoo W.J. (2020). Functional outcomes of hip arthroscopy for pediatric and adolescent hip disorders. Clin. Orthop. Surg..

[15] Sevencan A., Ucpunar H., Ozyalvac O.N., Akpinar E., Bayhan A.I., Yildirim T. (2022). Multivariate analysis of the predictive value of asymmetric skin creases in diagnosis of decentralized developmental dysplasia of the hip. J. Pediatr. Orthop. B.

[16] Groarke P.J., McLoughlin L., Whitla L., Lennon P., Curtin W., Kelly P.M. (2017). Retrospective multicenter analysis of the accuracy of clinical examination by community physicians in diagnosing developmental dysplasia of the hip. J. Pediatr..

[17] Ömeroglu H., Tatlici E., Köse N. (2020). Significance of asymmetry of groin and thigh skin creases in developmental dysplasia of the hip revisited: Results of a comparative study. J. Pediatr. Orthop..

[18] Kyung B.S., Lee S.H., Jeong W.K., Park S.Y. (2016). Disparity between clinical and ultrasound examinations in neonatal hip screening. Clin. Orthop. Surg..

[19] Cunningham K., Beningfield S., Moulton A., Maddock C. (1984). A clicking hip in a newborn baby should never be ignored. Lancet.

[20] Li Y., Canavese F., Liu Y., Wu J., Li J., Yuan Z., Zhou Q., Liu Y., Chen W., Xu H. (2022). Does a Graf Type-I Hip Justify the Discontinuation of Pavlik Harness Treatment in Patients with Developmental Dislocation of the Hip?. Children.

[21] De Pellegrin M., Damia C.M., Marcucci L., Moharamzadeh D. (2021). Double Diapering Ineffectiveness in Avoiding Adduction and Extension in Newborns Hips. Children.

[22] Graf R. (2007). Hip sonography: 20 years experience and results. Hip Int..

[23] Õmeroğlu H. (2014). Use of ultrasonography in developmental dysplasia of the hip. J. Child. Orthop..

[24] Lussier E.C., Sun Y.-T., Chen H.-W., Chang T.-Y., Chang C.-H. (2019). Ultrasound screening for developmental dysplasia of the hip after 4 weeks increases exam accuracy and decreases follow-up visits. Pediatr. Neonatol..

[25] Graf R. (2017). Hip sonography: Background; technique and common mistakes; results; debate and politics; challenges. Hip Int..

[26] Liu B., Hu X., Li L., Gao S. (2022). The Association of Asymmetric Skinfolds and the Diagnosis of Developmental Dysplasia of the Hip in Infants. Adv. Neonatal Care.

[27] Ando M., Gotoh E. (1990). Significance of inguinal folds for diagnosis of congenital dislocation of the hip in infants aged three to four months. J. Pediatr. Orthop..

[28] Louer C.R., Bomar J.D., Pring M.E., Mubarak S.J., Upasani V.V., Wenger D.R. (2019). Should paediatricians initiate orthopaedic hip dysplasia referrals for infants with isolated asymmetric skin folds?. J. Child. Orthop..

[29] Şenaran H., Özdemir H.M., Ögün T.C., Kapicioglu M.S. (2004). Value of limited hip abduction in developmental dysplasia of the hip. Pediatr. Int..

[30] Treiber M., Korpar B., Sirše M., Merc M. (2021). Early neonatal universal ultrasound screening for developmental dysplasia of the hip: A single institution observational study. Int. Orthop..

[31] Husum H.C., Bach Hellfritzsch M., Maimburg R.D., Henriksen M., Lapitskaya N., Møller-Madsen B., Rahbek O. (2022). Pubo-Femoral Distances Measured Reliably by Midwives in Hip Dysplasia Ultrasound. Children.

[32] Para A., Batko B., Ippolito J., Hanna G., Edobor-Osula F. (2021). Developmental Dysplasia of the Hip: How Does Social Media Influence Patients and Caregivers Seeking Information?. Children.

[33] Taylor I.K., Burlile J.F., Schaeffer E.K., Geng X., Habib E., Mulpuri K., Shea K.G. (2020). Developmental dysplasia of the hip: An examination of care practices of pediatric orthopaedic surgeons in North America. J. Pediatr. Orthop..

[34] Nie K., Rymaruk S., Paton R. (2017). Clicky hip alone is not a true risk factor for developmental dysplasia of the hip. Bone Joint J..

[35] Jari S., Paton R., Srinivasan M. (2002). Unilateral limitation of abduction of the hip. A valuable clinical sign for DDH?. J. Bone Joint Surg. Br..

[36] Swarup I., Penny C.L., Dodwell E.R. (2018). Developmental dysplasia of the hip: An update on diagnosis and management from birth to 6 months. Curr. Opin. Pediatr..

[37] Atalar H., Günay C., Atik O.Ş. (2021). Is treatment termination safe in developmental dysplasia of the hip following ultrasonographic normalization?. Jt. Dis. Relat. Surg..

